# A magnetic resonance nanoprobe with STING activation character collaborates with platinum-based drug for enhanced tumor immunochemotherapy

**DOI:** 10.1186/s12951-021-01158-y

**Published:** 2021-12-11

**Authors:** Jiali Li, Shichao Li, Yang Li, Guanjie Yuan, Yaqi Shen, Yang Peng, Li Kong, Conglian Yang, Zhiping Zhang, Zhen Li

**Affiliations:** 1 Department of Radiology, Tongji Hospital, Tongji Medical College, Huazhong University of Science and Technology, No. 1095 Jiefang Avenue, Wuhan, People’s Republic of China; 2 Tongji School of Pharmacy, Huazhong University of Science and Technology, Wuhan, 430030 People’s Republic of China; 3 Hubei Engineering Research Center for Novel Drug Delivery System, Huazhong University of Science and Technology, Wuhan, 430030 People’s Republic of China

**Keywords:** Manganese, STING activation, Platinum-based chemotherapeutics, Immunochemotherapy, Anti-tumor

## Abstract

**Background:**

Immunochemotherapy is a potent anti-tumor strategy, however, how to select therapeutic drugs to enhance the combined therapeutic effect still needs to be explored.

**Methods and results:**

Herein, a magnetic resonance nanoprobe (MnP@Lip) with STING (Stimulator of INterferon Genes) activation character was synthesized and co-administered with platinum-based chemotherapeutics for enhanced immunochemotherapy. MnP@Lip nanoparticles was prepared by simple fabrication process with good reproducibility, pH-sensitive drug release behavior and biocompatibility. In vitro experiments elucidated that Mn^2+^ can promote the polarization of M0 and/or M2 macrophages to M1 phenotype, and promote the maturation of BMDC cells. Upon Mn^2+^ treatment, the STING pathway was activated in tumor cells, mouse lung epithelial cells, and immune cells. More importantly, anti-tumor experiments in vivo proved that MnP@Lip combined with platinum-based chemotherapeutics increased T cells infiltration in the tumor microenvironment, and inhibited tumor growth in the orthotopic therapeutic and postoperative tumor models.

**Conclusions:**

This kind of therapeutic strategy that combined MnP@Lip nanoparticles with platinum-based chemotherapeutics may provide a novel insight for immunochemotherapy.

**Graphical Abstract:**

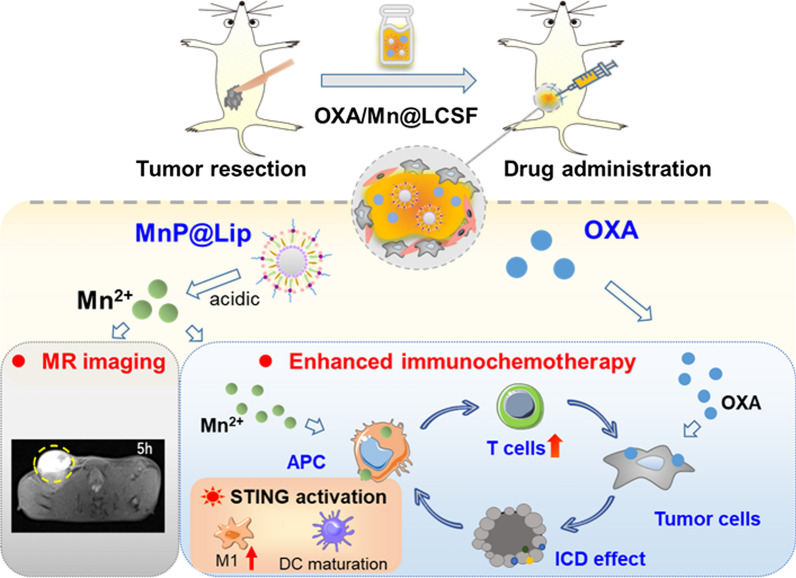

**Supplementary Information:**

The online version contains supplementary material available at 10.1186/s12951-021-01158-y.

## Introduction

Global Cancer Statistics 2020 indicated that cancer was the greatest cause of human deaths, and this warned us that the pace of cancer prevention and treatment must be accelerated [1]. MR imaging is an indispensable part during tumor diagnosis and treatment. Among MRI contrast agents in researches, manganese (Mn^2+^), a trace element to sustain life normal function, is a commonly used imaging probe [2, 3]. Recently, researches have pointed out that Mn^2+^ can activate stimulator of interferon genes (STING) pathway and played a crucial role in virus defense [4]. The recognized predominant mechanism was that Mn^2+^ can increase the sensitivity of cGAS to the cytosolic DNA in one hand, and can enhance the binding affinity of cGAMP to STING protein on the other hand [4–6]. Consequently, the downstream signaling pathway is strongly activated, and cytokines, especially for IFN-β, were produced, triggering a series of cascade reactions. Additionally, Mn^2+^ also performed excellent in activating anti-tumor immune response [5, 6]. Antigen-presenting cells can be effectively activated by Mn^2+^ through STING related pathway. However, there still exist safety concern and targeting problem for in vivo direct use of free Mn^2+^ ions [7]. Compared with free Mn^2+^ ions, nanoparticle modification can increase the retention time of Mn^2+^ in the tumor and improve biosafety in vivo [7]. Importantly, sustained release of Mn^2+^ from enable the maximization activation of STING pathway, thus leading to potent tumor killing ability [8–10]. Hence, it is of great significance to construct a nanoprobe with acidic response and sustained release behavior for fully exploiting the MR imaging and STING activation functions of Mn^2+^.

In tumor treatment, inadequate immune activation may be a critical factor that leads to treatment failure [11, 12]. Combination immunotherapy with other classic therapies (such as surgery, chemotherapy, and radiotherapy) is becoming mainstream in cancer treatment [13, 14]. Low dose of chemotherapy has now been confirmed to activate the immune response, and thence enhance the effectiveness of immunotherapy [15–17]. For example, studies have shown that chemotherapeutic drug doxorubicin can improve tumor immunogenicity while killing tumor cells, and can significantly increase the anti-tumor effect when combined with immune checkpoint inhibitors [18]. In addition, the combination of chemotherapeutic drug paclitaxel and cytokine IL-12 showed enhanced activation of T cells and NK cells [19]. Through in-depth study on the STING activation mechanism of Mn^2+^, we found that Mn^2+^ could better activate the downstream pathway when binds with dsDNA. Promisingly, platinum-based chemotherapeutics enable to promote double-stranded DNA (dsDNA) production by damaging DNA of tumor cells [20, 21]. Based on the above hypothesis, the combination of Mn^2+^ nanoprobes and platinum-based chemotherapeutics may achieve synergistic anti-tumor effect, and realize the integration of diagnosis and treatment.

**Scheme 1:**
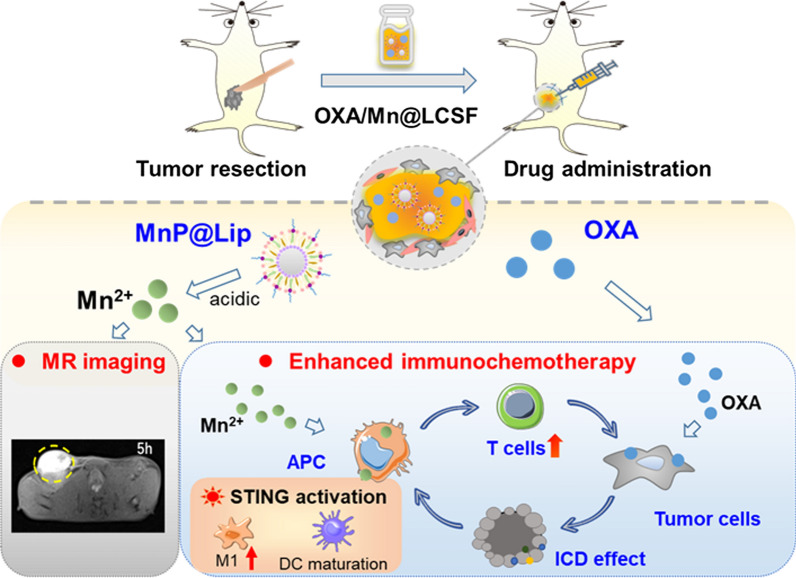
An injectable gel system co-loaded with MnP@Lip nanoparticles and OXA for MR imaging and enhanced immunochemotherapy. MnP@Lip nanoparticles exert the MR imaging capability and can activate STING pathway in macrophage and DC. OXA can induce immunogenic cancer cell death and synergistically elicit the immune system with MnP@Lip nanoparticles to prevent tumor recurrence

Herein, a magnetic resonance nanoprobe (MnP@Lip) with STING activation character was synthesized and co-administered with platinum-based chemotherapeutics for enhanced immunochemotherapy (Scheme 1). This study first verified the immune activation ability of Mn^2+^ in different cells and studied the action mechanism. Then, MnP@Lip nanoparticles were constructed and the physicochemical properties and MR imaging capability were characterized. Finally, the combined treatment efficiency of MnP@Lip nanoparticles and platinum-based chemotherapeutics was demonstrated in 4T1 and MC38 tumor models. Additionally, in the model of postoperative tumor recurrence, a simple liquid crystal gel formation system (LCFS) containing oxaliplatin (OXA) and MnP@Lip nanoparticles was shown to inhibit tumor recurrence effectively. This kind of therapeutic strategy combined MnP@Lip nanoparticles with platinum-based chemotherapeutics may provide a novel insight for immuno-chemotherapy.

## Materials and methods

### Materials and reagents

Manganese chloride (MnCl_2_), Na_2_HPO_4_.12H_2_O and cyclohexane were obtained from Aladdin®, China. CO-520 was purchased from Sigma-Aldrich, USA. Dioleoyl phosphatidic acid (DOPA), cholesterol, 1,2-dioleoyl-sn-glycero-3-phosphoethanolamine-N-[methoxy(polyethylene, glycol)-2000] and 1,2-dioleoyl-snglycero-3-phosphocholine (DOPC) were bought from Avanti Polar Lipids, Inc, USA. Fetal bovine serum (FBS) and Dulbecco’s modified Eagle’s medium (DMEM) were bought from Hyclone, USA. The 3-(4, 5-dimethyl-thiazol-2-yl)-2,5-diphenyltetrazolium bromide (MTT) was purchased from BioSharp (South Korea). All antibodies for flow cytometric analysis were bought from BD Biosciences (USA). cGAS (D3O8O), pSTING(Ser366), STING(D2P2F), pNF-кB p65 (Ser536), NF-кB p65 (L8F6), pIRF3 (Ser396), IRF3 (D83B9), GAPDH were purchased from Cell Signaling Technology (USA).

### Cell lines and animals

MC38 and 4T1 cells were bought from Cell Bank of Chinese Academy of Science (Shanghai, China) and were separately cultured in RPMI and DMEM medium comprising 10% Fetal Bovine Serum (FBS) and antibiotics (1% streptomycin and 100 IU/mL penicillin). Humidified atmosphere and 5% CO_2_ were needed for the culture environment. C57BL/6 (female, 5–6 weeks) and BALB/c mice (female, 5–6 weeks) were purchased from Hubei Provincial Center for Disease Control and Prevention (Wuhan, China), and the experimental manipulation on mice was all performed according to principles of Animal Care and Use Committee in Huazhong University of Science and Technology.

### Induced polarization of macrophage by Mn^2+^

#### Polarization and STING activation of Mn^2+^ on Raw 264.7 cells

Raw 264.7 cells were seeded in a 24-well plate (2 × 10^5^ cells/well). After incubation overnight, the supernatant was discarded. Blank DMEM medium or different MnCl_2_ concentrations (0, 0.1, 0.2, and 0.4 mM) were added into cells for culturing additional 24 h. Concentrations of IFN-β in the supernatant were tested by a bioluminescent ELISA kit (LumiKine™ Xpress mIFN-β 2.0), and flow cytometer was used to examine the CD86 expression level of Raw 264.7 cells.

To test the ROS production, Raw 264.7 cells were seeded in a 24-well plate (1 × 10^5^ cells/well). After incubation overnight, the supernatant was discarded. The cells were washed once with PBS, then blank DMEM or different MnCl_2_ concentrations (0, 0.1, 0.2, and 0.4 mM) were added into for culturing additional 6 h. The assay kit (Beyotime Institute of Biotechnology, China) and flow cytometer was used to test the ROS production.

#### STING activation and polarizing bone marrow derived macrophage (BMDM) from M0 to M1 phenotype by Mn^2+^

BMDM cells were obtained by referring to previous methods [22]. Bone marrow progenitor cells were extracted from the leg bones of C57 mice, and were cultured in RPMI-1640 medium that included 20% L929 cultured medium supernatant (vol/vol), 10% FBS, 1% streptomycin and 100 IU mL^−1^ penicillin. L929 medium could help to induce bone marrow progenitor cells differentiate into BMDM cells. After culturing for 5 days, immature BMDM cells (M0) were collected for further use.

M0 macrophages were seeded in a 24-well plate (1 × 10^6^ cells/well), and the blank RPMI-1640 or different MnCl_2_ concentrations (0, 50, 100, 200 and 400 μM) were added for stimulation. After 24 h, the supernatant was collected to test the production of IFN-β, and the cells were harvested to examine the CD80 expression level by a flow cytometer. Another group of BMDM cells (2 × 10^6^ cells/well, 6-well plate) that were treated with MnCl_2_ (0, 50, 100, 200 and 400 μM) were lysed to obtain protein, and the expression of cGAS, pSTING/STING, pNF-кB p65/NF-кB p65 and pIRF3/ IRF3 were tested by western blot.

#### Polarizing BMDM from M2 to M1 by Mn^2+^

M0 macrophages were obtained according to the procedures described above and seeded in a 24-well plate ((1 × 10^6^ cells/well). IL-4 (20 ng/mL) was added and reacted 24 h to induce M0 to M2 phenotype. Next, the medium was replaced with different concentrations of MnCl_2_ (0, 50, 100, 200 and 400 μM) to keep incubating for 24 h. Flow cytometer was used to examine the ratio of M1 and M2 macrophages.

### Maturation and STING activation of BMDC by Mn^2+^

BMDC cells were obtained by referring to previous methods [23]. Bone marrow progenitor cells were extracted from the leg bones of C57 mice, and were cultured in RPMI-1640 medium that included 20 ng/mL GM-CSF, 10% FBS, 1% streptomycin and 100 IU/mL penicillin. GM-CSF could help to induce bone marrow progenitor cells differentiate into BMDC cells. After culturing for 7 days, immature BMDC cells were collected for further use.

Immature BMDC cells were seeded in a 24-well plate ((1 × 10^6^ cells/well), and LPS and different MnCl_2_ concentrations (0, 50, 100, 200 and 400 μM) in RPMI-1640 medium were added into for incubation. After 24 h, flow cytometer was used to examine the cell phenotype. Additionally, immature BMDC cells (2 × 10^6^ cells/well, 6-well plate) treated with MnCl_2_ (0, 50, 100, 200 and 400 μM) were used to test the expression of cGAS, pSTING/STING, pNF-кB p65/NF-кB p65 and pIRF3/ IRF3 by western blot.

### STING activation in tumor cells and mouse lung epithelial (MLE) cells by Mn^2+^

MC38 and MLE-12 cells (5 × 10^5^ cells/well, 6-well plate) were inoculated into 6-well plates. After 12 h, the supernatant was replaced by blank medium including different concentrations of MnCl_2_ (0, 50, 100, 200 and 400 μM) to keep incubating for 24 h. Then cells were collected and lysed to obtain protein, and the expression of cGAS, pSTING/STING, pNF-кB p65/NF-кB p65 and pIRF3/ IRF3 were tested by western blot.

MLE-12 cells were respectively seeded in a 24-well plate (2 × 10^5^ cells/well). DMEM medium or different MnCl_2_ concentrations were added into cells for culturing 24 h. Concentrations of IFN-β in the supernatant were tested by a bioluminescent ELISA kit (LumiKine™ Xpress mIFN-β 2.0).

### In vitro cytotoxicity analysis of Mn^2+^ in RAW 264.7, BMDC, MC38 and MLE cells

RAW264.7 (2 × 10^4^ cells/well), BMDC (2 × 10^4^ cells/well), MC38 cells (1 × 10^4^ cells/well) and MLE cells (1 × 10^4^ cells/well) were respectively cultured in a 96-well plate for 24 h. The supernatant was replaced by blank medium including different concentrations of MnCl_2_ to keep incubating for 24 h. Then the cell viability was tested by MTT assay (λ = 490 nm) using a microplate reader (Multiskan, MK3, Thermo Fisher Scientific, Waltham, MA).

### Synthesis and characterization of MnP@Lip

The synthesis procedure mainly involved two parts. Part 1, solution A (2 mL CO-520/cyclohexane + 25 μL DOPA (20 mg/mL) + 50 μL Na_2_HPO_4_.12H_2_O (100 mM)) and solution B (2 mL CO-520/cyclohexane + 50 μLMnCl_2_ (500 mM)) were stirred separately for 30 min. Under magnetic stirring, solution B was added dropwise into solution A. After stirring for 2 h, absolute ethanol (4 mL) was used to demulsify. The non-nanoparticles of mixed solution were removed by centrifugation (13,000 × g for 15 min). The precipitate (MnP nanoparticles) was collected and dried by nitrogen gas. Part 2, the phospholipids dissolved in chloroform, consisting of DOPC (40 μL, 20 mM), cholesterol (40 μL, 20 mM) and DSPE-PEG2000 (10 μL, 20 mM), were added into above precipitate. After the removal of chloroform by rotary evaporation, phosphate buffer saline (PBS) was added for hydration to form MnP@Lip nanoparticels.

The morphology of MnP nanoparticles and MnP@Lip was confirmed through transmission electron microscope (TEM, JEM-1230, Japan). The hydrodynamic diameter, zeta potential and stability of MnP@Lip were measured and recorded using dynamic light scattering (DLS, Zeta Plus, Brookhaven Instruments, USA). The release behavior of Mn^2+^ from MnP@Lip in different pH ABS (5.5 and 7.4) were evaluated using dialysis bags (molecular weight cutoff, 3500–5000). The sample outside the dialysis bag was collected at specific time points, and the released amount of Mn^2+^ was measured using flame atomic absorption spectroscopy (SpectrAA-240FS, Varian, Palo Alto, CA, USA). The r_1_ relaxivity of MnP@Lip at different pH values (5.5 and 7.4 ABS) was evaluated by a 3 T MRI scanner (123.3 MHz.). The scanning scheme and calculation method are consistent with previous study [24].

### In vivo MR imaging

4T1 orthotopic tumor model was established to verify in vivo MR imaging ability of MnP@Lip, by using 3 T MRI scanner equipped with specific mice coil. 4T1 cells (1 × 10^6^/150 μL) were inoculated to left lower mammary pad of BALB/c mice, then MnP@Lip (50 μL, 1 mg/kg Mn) was intratumorally injected after 7 days. MR imaging (T1WI) was performed at pre and 0.5 h, 1 h, 2 h, 5 h, 24 h after MnP@Lip injection. The ratio of tumor and normal tissue signal was calculated to objectively present imaging ability. Detailed MRI scanning parameters are shown in Additional file 1: Table S1.

### Synergistic anti-tumor efficacy study

Multiple tumor models were established to validate the synergistic anti-tumor efficacy of MnP@Lip and platinum-based drugs, including 4T1 orthotopic tumor model, MC38 subcutaneous tumor model and 4T1 incomplete tumor resection model.

4T1 cells (1 × 10^6^/150 μL) were inoculated to left lower mammary pad of BALB/c mice to establish the orthotopic tumor model. When tumor volume reached 50–100 mm^3^, the mice were randomized into PBS group, MnP@Lip group, oxaliplatin (OXA) group and OXA + MnP@Lip group. Each group of mice received matching drug or PBS treatment for four times (with the interval of 3 days), such as PBS (i.t. 100 μL), MnP@Lip (i.t. 1 mg/kg of Mn, 100 μL) and OXA (i.p. 3 mg/kg, 100 μL). Tumor size (Volume = 0.5 × lengh × wide^2^) and weight of the mice during the experimentation were documented every 3 days. At the termination of this research, the mice serum was obtained to evaluate hepatic and renal function, then tumors were collected to perform immunohistochemistry and immunofluorescence assay.

MC38 cells (2.5 × 10^5^/150 μL) were inoculated to the right armpit of C57 mice to establish the subcutaneous tumor model. When tumor volume reached 50–100 mm^3^, the mice were randomized into PBS group, cisplatin (Pt) group, and Pt + MnP@Lip group. Each group of mice received matching drug or PBS treatment four times (with the interval of 3 days), such as PBS (i.t. 100 μL), Pt (i.p. 3 mg/kg, 100 μL) and MnP@Lip (i.t. 1 mg/kg of Mn, 100 μL). Tumor size (Volume = 0.5 × length × wide^2^) and weight of the mice during the experimentation were documented every 3 days. At the termination of this research, tumors were collected, pictured and weighted.

To establish incomplete tumor resection model, 4T1 cells (1 × 10^6^/150 μL) were inoculated to left lower mammary pad of Balb/c mice, then tumors were surgically excised after 10 days of transplantation, and we tried to make sure that the remaining tumors were similar in size. The postoperative mice were randomized into PBS group, OXA group, OXA + MnCl_2_ group and OXA + MnP@Lip group. Each group of mice received matching drug or PBS treatment one time, such as PBS (i.t. 100 μL), MnP@Lip (i.t. 1 mg/kg of Mn, 100 μL), MnCl_2_ (i.t. 1 mg/kg of Mn, 100 μL) and OXA (i.p. 3 mg/kg, 100 μL). The above-mentioned drugs and PBS were all dispersed in a liquid crystal gel formation system for stable and sustained drug release according to previous study in our lab [25]. The rheology experiments of LCFS precursor and LCFS gel was tested by Dynamic shear rheometer (Kinexus Rotational Rheometer, Malvern Instruments, Malvern, UK). During the experimentation, tumor growth and weight of mice were observed and recorded. At the termination of this research, tumors were collected to perform immunohistochemistry and immunofluorescence assay.

### In vivo safety analysis

In addition, safety study of combined treatment of MnP@Lip nanoparticles and OXA was performed. Briefly, PBS, OXA, OXA + MnCl_2_, and OXA + MnP@Lip were separately dispersed in LCFS and then they were separately injected under the skin of the mice. The body weight of mice was recorded for 7 consecutive days, blood samples were collected for liver and kidney function detection, and the HE staining of major tissues and organs were also investigated.

### Statistical analysis

The statistical difference between groups was evaluated by SPSS software (version 23.0). The two-tailed Student's *t* test or One-way ANOVA (including Tukey post-hoc test) was used for data satisfying normality and homogeneity of variance. The Mann–Whitney test or Kruskal–Wallis test (including Bonferroni post-hoc correction) was used to compare data that satisfy non-normality or heterogeneity variance. In figure captions, one star (*) means *p* < 0.05, two (**) means *p* < 0.01 and three (***) means *p* < 0.001, and “ns” means no statistically significant.

## Results and discussion

### Macrophage polarization effect induced by Mn^2+^

STING pathway was found to be activated by Mn^2+^ in multiple perspectives through increasing the affinity of cGAS to dsDNA, promoting cGAMP production and thereby inducing the secretion of type-I IFN [4, 5]. Additionally, cGAMP-STING activation was reported to produce a large number of proinflammatory macrophages [26]. To confirm whether Mn^2+^ could induce macrophage polarization while activating STING pathway, this study exposed macrophages to different concentrations Mn^2+^ and detected the polarization effect of Mn^2+^.

**Fig. 1:**
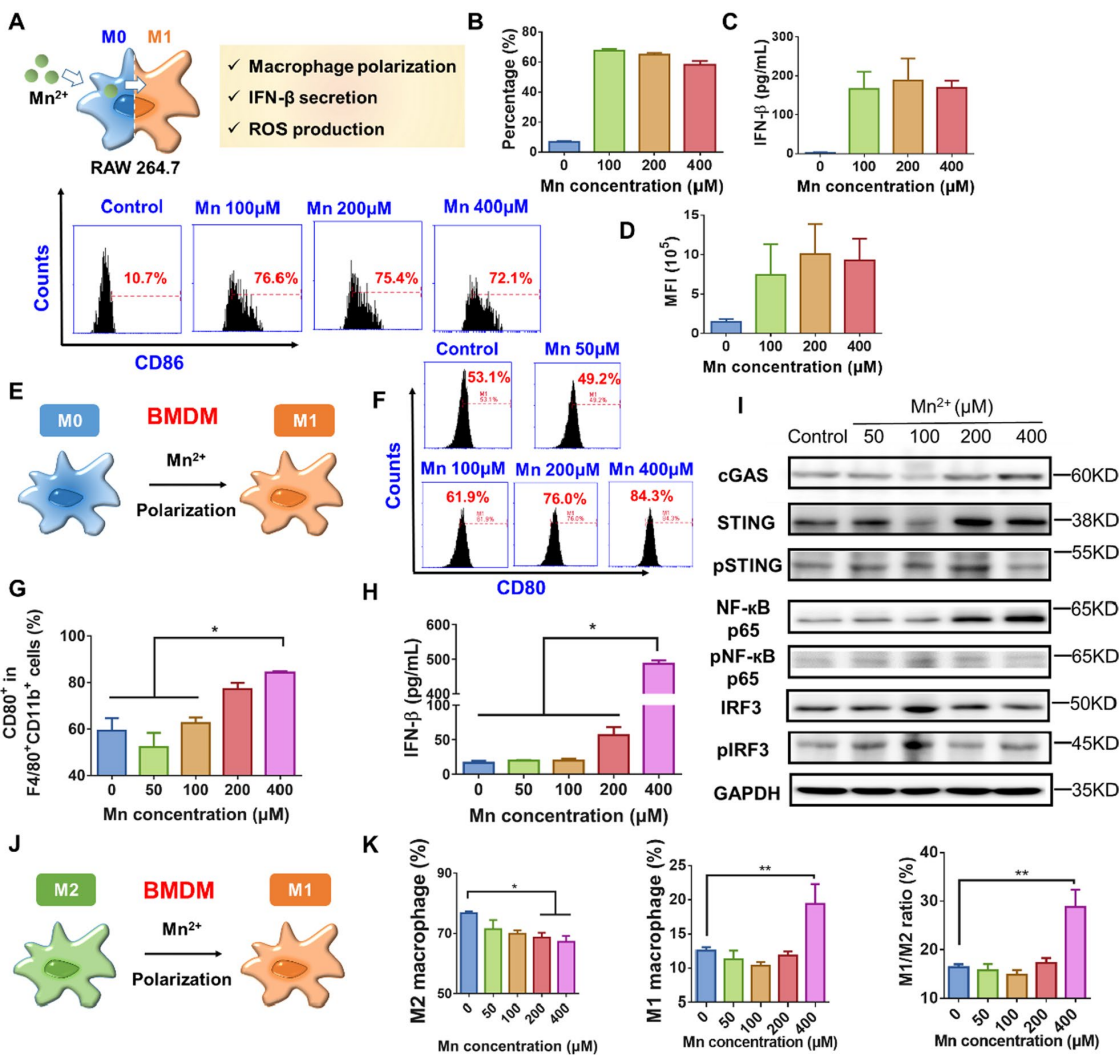
Macrophage polarization effect induced by Mn^2+^. **A** RAW 264.7 cells polarization induced by Mn^2+^. **B** The percentage of CD11b^+^CD86^+^ in RAW 264.7 cells treated with different concentrations of Mn^2+^ (n = 3). **C** IFN-β generation of RAW 264.7 cells that were treated with different concentrations of Mn^2+^_._ (n = 3). **D** Mean fluorescence intensity (MFI) of ROS generation in RAW 264.7 cells that were treated with different concentrations of Mn^2+^ (n = 3). **E** Schematic diagram of polarization promoting effect of Mn^2+^ on BMDM cells from M0 to M1 phenotype. **F** and **G** The representative flow cytometry (FCM) and quantitative results of BMDM cells polarization effect induced by Mn^2+^ (n = 3). **H** IFN-β generation of BMDM cells that were treated with different concentrations of Mn^2+^(n = 3). **I** cGAS, pSTING/STING, pNF-кB p65/NF-кB p65 and pIRF3/IRF3 protein expression of BMDM cells that were treated with different concentrations of Mn^2+^. **J** Schematic diagram of polarization promoting effect of Mn^2+^ on BMDM cells from M2 to M1 phenotype. **K** The ratio of M1 and M2 macrophages after treating with different concentrations of Mn^2+^ (n = 3)

We first investigated the polarization effect of Mn^2+^ on immortalized Raw 264.7 cells. The results (Fig. 1A and B) indicated that 0.1, 0.2 and 0.4 mM of Mn^2+^ all enabled the enhanced CD86 expression on Raw 264.7 cells, which demonstrated Raw 264.7 cells were transformed into M1 type under the effect of Mn^2+^. Moreover, 0.1 mM seemed to be sufficient for Mn^2+^ to induce the polarization effect of Raw 264.7 cells. This study also explored the secretion of IFN-β and ROS in macrophages after co-culturing with Mn^2+^ for 24 h. IFN-β is an important indicator of the activation of STING pathway. The results of IFN-β (Fig. 1C) indicated that all concentrations of Mn^2+^ were able to stimulate Raw 264.7 cells to produce IFN-β. Figure 1D indicated that all concentration of Mn^2+^ were able to promote the generation of ROS. In addition, cell safety test showed that Raw 264.7 cells showed good tolerance to Mn^2+^ (Additional file 1: Figure S1). In summary, this part data proved that Mn^2+^ stimulation can promote the production of ROS and IFN-β in Raw 264.7 cells, and increase the expression of CD86 to transform Raw 264.7 cells into pro-inflammatory M1-type macrophages.

### Polarizing BMDM from M0 to M1

The results of Mn^2+^ on Raw 264.7 cells encouraged us to continue to explore whether Mn^2+^ has a similar effect on BMDM cells. This study exposed BMDM cells to different concentrations of Mn^2+^ for 24 h, and the expression level of CD80 and secretion of IFN-β were tested (Fig. 1E). The results of flow cytometry analysis (Fig. 1F–H) showed that Mn^2+^ significantly promoted the expression of CD80 on BMDM cells (F4/80^+^CD11b^+^) in a dose-dependent way, and the expression difference between control group and 200 or 400 μM group were statistically significant. In addition, the study on cGAS, pSTING/STING, pNF-кB p65/NF-кB p65 and pIRF3/ IRF3 protein expression in BMDM cells stimulated by Mn^2+^ was carried out. The results (Fig. 1I) demonstrated that the protein expression in BMDM cells induced by Mn^2+^ both enhanced. Furthermore, IFN-β concentration secreted BMDM cells sharply increased when drug concentration came to 400 μM. Above results were similar to those of Mn^2+^ on RAW 264.7 cells. In summary, the use of Mn^2+^ could promote the expression of cGAS, pIRF3, NF-κB p65, IFN-β secretion and CD80 level in BMDM cells, thus activate STING pathway and polarize BMDM cells to proinflammatory M1 phenotype.

### Polarizing BMDM from M2 to M1

M2 macrophages constitute an important part of the immunosuppressive tumor microenvironment, and M2-to-M1 repolarization has significant therapeutic value[27]. Therefore, based on the results that M0 macrophages were polarized to M1 phenotype by Mn^*2*+^, this study further explored that whether Mn^2+^ can polarize M2 macrophages to M1 phenotype and improve the immunosuppressive microenvironment in tumor (Fig. 1J). M2 macrophages were exposed to different concentrations of Mn^2+^ for 24 h, and the proportion of M1/M2 macrophages was tested by flow cytometry. As shown in Fig. 1K, unlike M1 macrophages which only changed at 400 μM, the proportion of M2 macrophages gradually decreased with the enhancement of Mn^2+^. M1 macrophage is a pro-inflammatory phenotype with anti-tumor activity, while M2 macrophage is an immunosuppressive cell phenotype that promotes tumor progression [28, 29]. Hence, all the above results indicated that Mn^2+^ can polarize M2 macrophages into M1 phenotypes, which is of great significance to improve the immunosuppressive microenvironment.

### STING activation effect of Mn^2+^ in different cells

#### DC cells

**Fig. 2:**
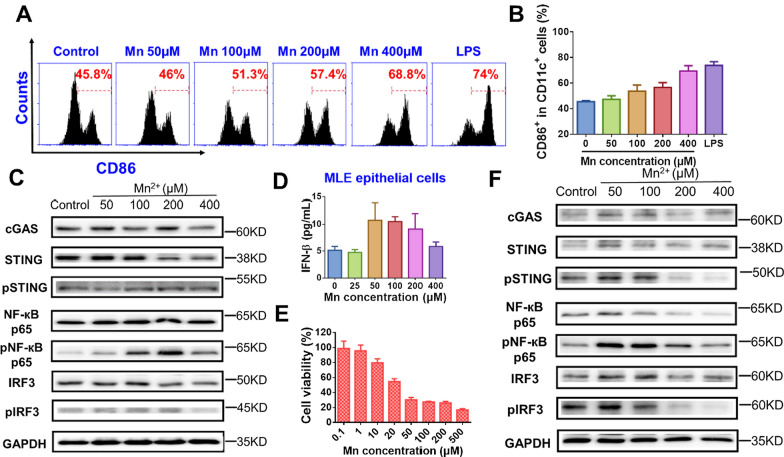
STING activation effect of Mn^2+^ in different cells. **A** and **B** the representative and quantitative maturation of BMDC cells induced by different concentrations of Mn^2+^ (n = 3). **C** cGAS, pSTING/STING, pNF-кB p65/NF-кB p65 and pIRF3/IRF3 protein expression of MC38 cells that were treated with different concentrations of Mn^2+^. **D** IFN-β generation of MLE-12 cells treated with different concentrations of Mn^2+^ (n = 3). **E** Cell viability of Mn^2+^ on MC38 cells (n = 3). **F** cGAS, pSTING/STING, pNF-кB p65/NF-кB p65 and pIRF3/IRF3 protein expression of MLE cells that were treated with different concentrations of Mn^2+^

DC cells were the most efficient antigen-presenting cells, so this study investigated the maturation effect of Mn^2+^ on BMDC cells. As shown in Fig. 2A and B, similar to macrophages, Mn^2+^ exhibited a dose-dependent maturation effect on BMDC cells. The CD86 expression on BMDC was the highest with 400 μM Mn^2+^, and even was equivalent to stimulatory effect of LPS. Cell safety test showed that the survival rate of BMDC cells showed good tolerance to Mn^2+^ in the concentration range from 0.1 to 500 μM (Additional file 1: Figure S1). This indicates that Mn^2+^ had strong maturation effect on BMDC, thus can enhance the activation of innate immunity. In addition, the results of cGAS, pSTING/STING, pNF-кB p65/NF-кB p65 and pIRF3/ IRF3 protein expression in BMDC cells stimulated by Mn^2+^ was shown in Additional file 1: Figure S2.

#### MC38 and MLE-12 cells

The STING pathway plays a role in other cells. Therefore, in addition to immune cells, this study also explored the activation of STING by Mn^2+^ on tumor cells MC38 and pulmonary epithelial cells MLE-12. The results in Fig. 2C demonstrated that upon Mn^2+^ stimulation, most of the expression amount of cGAS, pSTING/STING, pNF-кB p65/NF-кB p65 and pIRF3/ IRF3 firstly enhanced, and then decreased with the enhancement of drug dosage. As presented in Fig. 2D, 50 μM Mn^2+^ maximized the IFN-β production of MLE-12 cells. This indicated that Mn^2+^ not only activated STING pathway in antigen-presenting cells, but also in MC38 and MLE-12 cells. The increased IFN-β may stimulate immune cells, enhance innate and adaptive immune responses, and finally enhance anti-tumor effects [30]. MTT assay results showed that high concentration Mn^2+^ showed certain cytotoxicity to MC38 and MLE cells (Fig. 2E and Additional file 1: Figure S1). The results in Fig. 2F showed the expression results of cGAS, pSTING/STING, pNF-кB p65/NF-кB p65 and pIRF3/ IRF3 in MLE cells, which demonstrated a similar tendency with that in MC38 cells. The protein expression in Fig. 2C and F was may be explained by the cytotoxicity of high Mn^2+^ concentration in MC38 and MLE cells.

## Synthesis and characterization of MnP@Lip

**Fig. 3:**
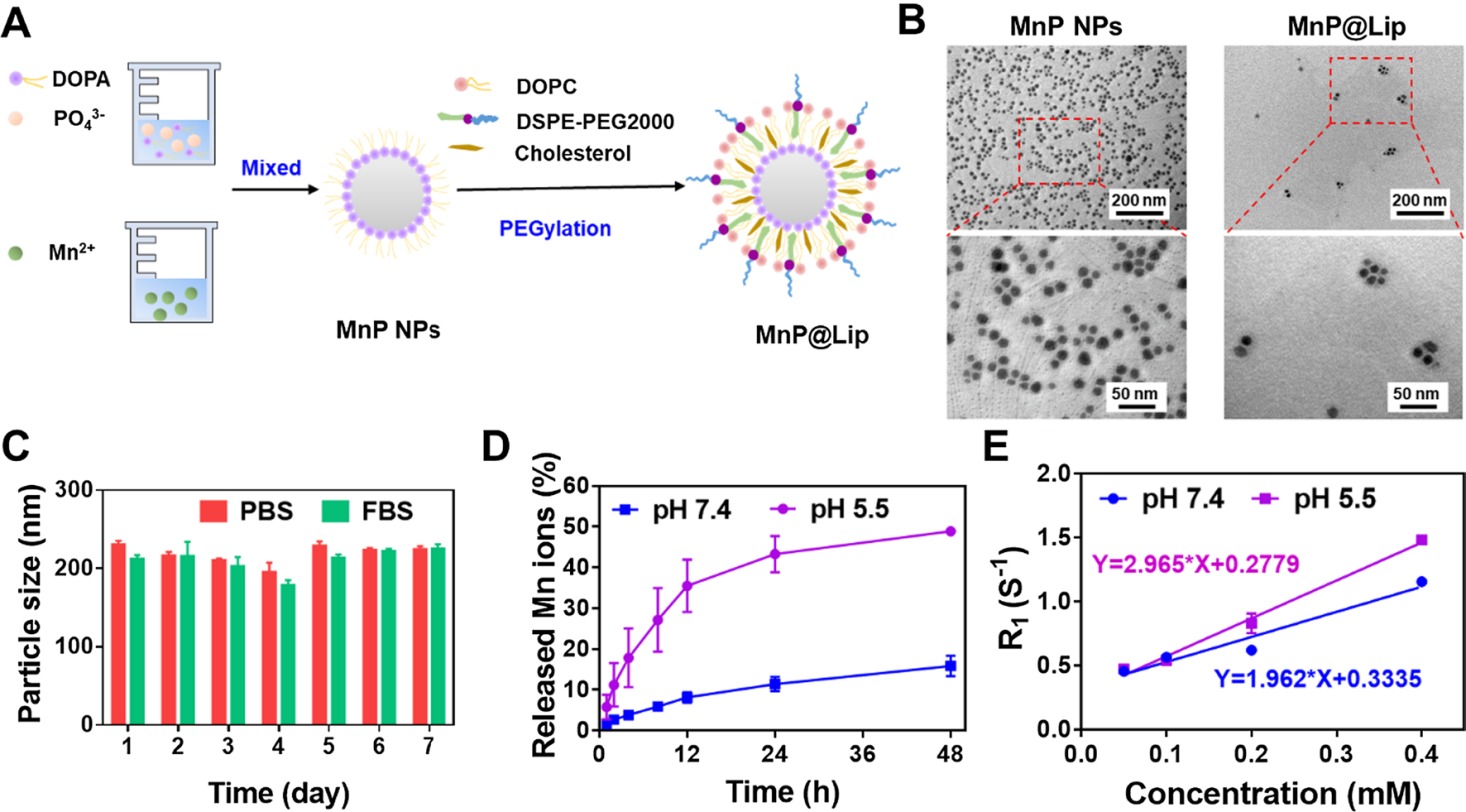
Preparation and characterization of MnP@Lip nanoparticles. **A** Schematic illustration of the preparation process of MnP@Lip. **B** TEM images of MnP NPs and MnP@Lip nanoparticles. **C** Stability analysis of MnP@Lip nanoparticles in PBS and FBS (n = 3). **D** Release profiles of MnP@Lip nanoparticles at different pH (n = 3). **E** Longitudinal relaxivity rate R1 measurement of MnP@Lip nanoparticles (n = 3)

MnP@Lip was manufactured by reverse microemulsion approach and the schematic illustration of preparation method was shown in Fig. 3A. Briefly, Na_2_HPO_4_.12H_2_O and MnCl_2_ in microemulsion interacted to form nanosized MnP nanoparticles. Then lipids composition including DOPA, cholesterol, DOPC and DSPE-PEG2000 were added to improve the biocompatibility and dispersity of MnP nanoparticles, forming MnP@Lip nanoparticles. TEM images showed that the morphology of MnP NPs and MnP@Lip were both spherical (Fig. 3B). Compared with MnP NPs, the surface of MnP@Lip nanoparticles was equipped with lipid bilayer self-assembled by DOPA and DOPC, which can increase the dispersity. Furthermore, cholesterol and DSPE-PEG2000 interspersed in the lipid bilayer could stabilize the nanoparticle structure and prolong the circulation time, respectively [31, 32]. The hydrodynamic diameter and zeta potential of MnP@Lip were 210.4 nm and −38.54 mV, respectively. For particle size, the hydrodynamic diameter measured by DLS was larger than that measured directly by TEM. The possible explanation was that DLS technique takes the aqueous layer (stern layer) with positive and negative charges into account, while TEM only determine the real diameter of the nanoparticles without the aqueous layer around it. This difference in nanoparticle size measured by DLS and TEM also exists in other studies [32, 33]. In addition, the particle size of MnP@Lip dispersed in PBS or FBS was successively measured for 7 days. The negligible change of particle size in Fig. 3C indicated the good stability of MnP@Lip. The pH-responsive release property of MnP@Lip was presented in Fig. 3D. Within 24 h, cumulative release amount of Mn^2+^ was approximately 10% in PBS (pH 7.4), yet it was about 40% in PBS (pH 5.5), indicating that more Mn^2+^ was released from MnP@Lip at lower pH. This proved that MnP@Lip was pH-sensitive, and Mn^2+^ was able to released intracellularly which in turn enhanced immunity response. To explore the MR imaging ability, the longitudinal relaxivity (r_1_) was estimated. As displayed in Fig. 3E, r_1_ at pH 5.5 (2.96 mM^–1^·s^–1^) was 1.5 fold compared with that at pH 7.4 (1.96 mM^–1^·s^–1^), which proved the MR imaging ability of MnP@Lip. The plots of signal *vs.* inversion times (TI) were shown in Additional file 1: Figure S3.

## In vivo MR imaging ability of MnP@Lip

**Fig. 4:**
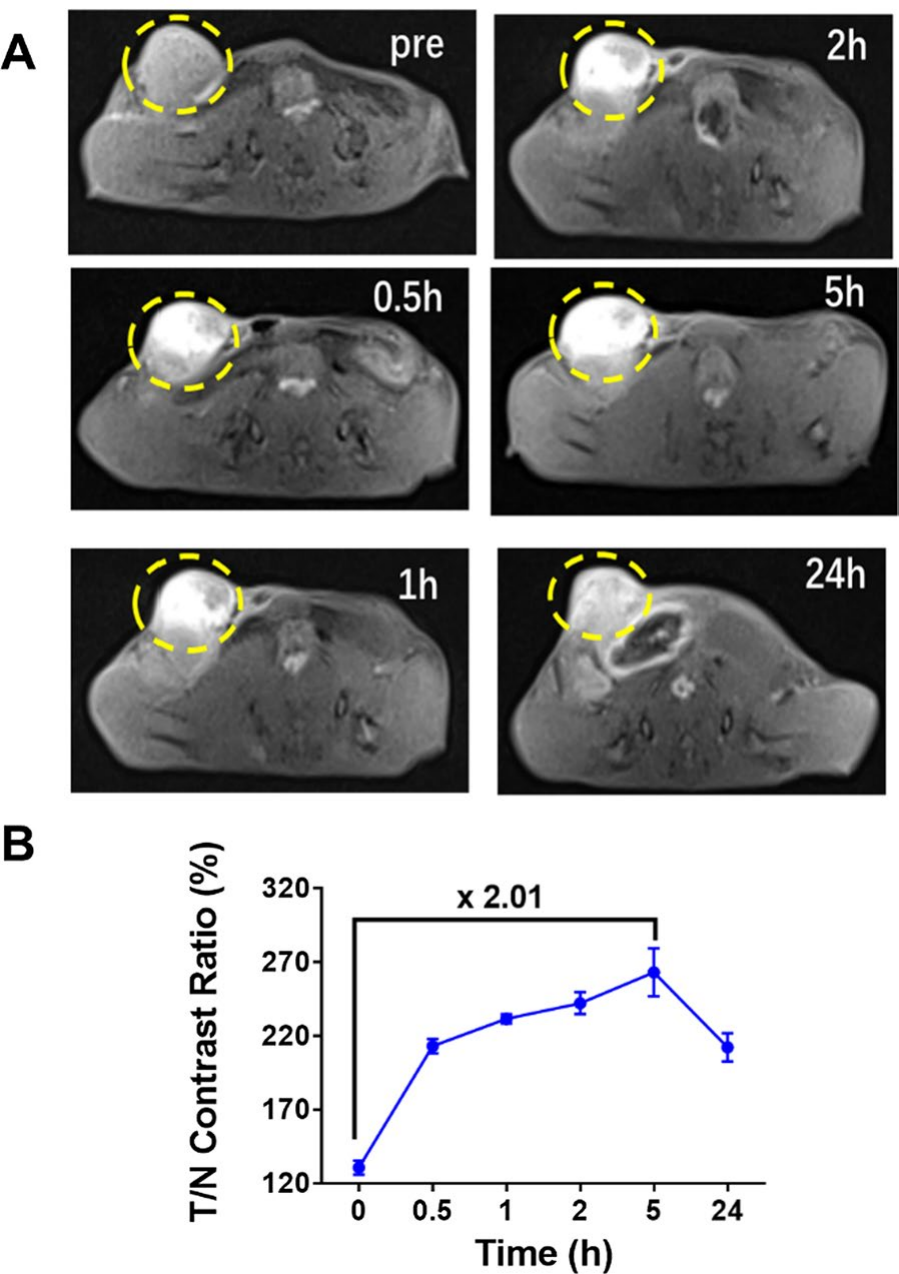
MRI imaging (T1WI) of 4T1 orthotopic tumor model after intratumoral injection of MnP@Lip (n = 3, Mn 1 mg/kg). **A** The representative MR images of tumor. The yellow circles indicate the location of the tumor. **B** The quantitative results of MRI signal intensity in tumor

Next, we evaluated the tumor MRI imaging ability of MnP@Lip in vivo. 4T1 orthotopic tumor model was used for imaging by using 3 T MRI scanner equipped with specific mice coil. As shown in Fig. 4, the MRI signal of tumor increased significantly at 0.5 h after MnP@Lip injection, and the signal intensity reached the peak at 5 h. In addition, even 24 h after injection, there was still significant MRI signal intensity in the tumor. Comprehensively, MnP@Lip was available for MR imaging.

## Synergistic anti-tumor efficacy study

Platinum-based chemotherapeutics can interfere with DNA replication by binding to the DNA strands, then cisplatin–DNA adducts are formed to destroy tumor cells [20, 21]. The effect of platinum-based chemotherapeutics caused the leakage of double strands DNA (dsDNA) to into the cytoplasm, and the increased amount of dsDNA were recognized by cGAS to activate STING pathway. Excitingly, Mn^2+^ can increase the sensitivity of cGAS to the cytosolic DNA. Given the verified the STING pathway activating effect of Mn^2+^ by in vitro experimentation, therefore, this study further explored the synergistic effect of MnP@Lip and platinum-based chemotherapeutics to activate immunity in multiple tumor models.

**Fig. 5:**
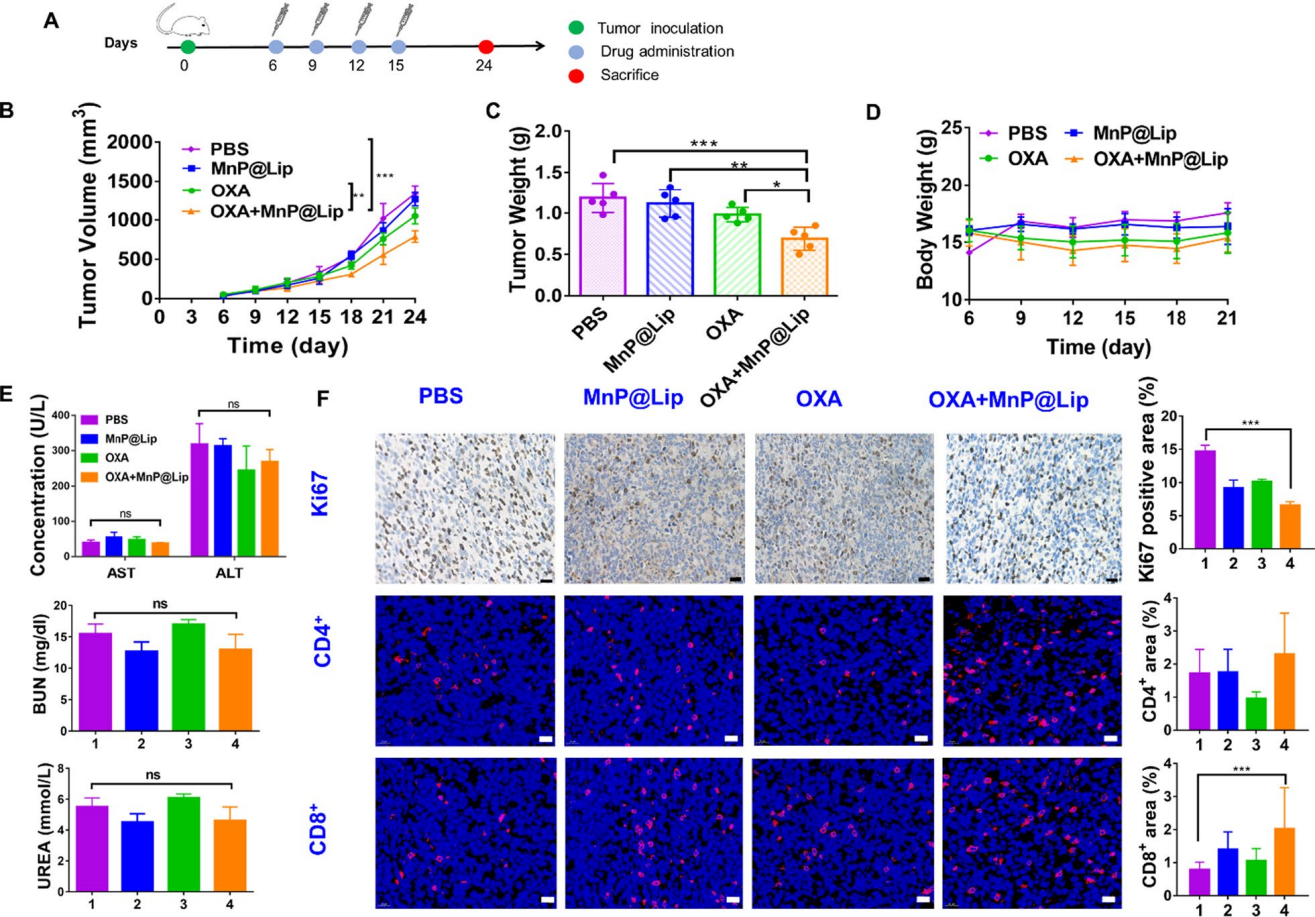
Anti-tumor effect of MnP@Lip combined with OXA in 4T1 orthotopic tumor model. **A** Treatment protocol of MnP@Lip combined with OXA in anti-tumor therapy. **B** Tumor growth curves of different treatment groups (n = 5). **C** Tumor weight change after receiving different treatments (n = 5). **D** Tumor weight change during the trial. **E** Effect of treatment on liver and kidney functions. **F** Immunohistochemical and immunofluorescence analysis of Ki67, CD8^+^ and CD4^+^ cells (n = 3). 1, 2, 3 and 4 represent PBS group, MnP@Lip group, OXA group and OXA + MnP@Lip group, respectively. The scale bars are 20 μm. The data of Figure B, Figure C, ASL, ALT and Ki67 satisfying normality, thus One-way ANOVA (including Tukey post-hoc test) or Student's t test was applied. The data of UREA, BUN, CD8^+^ and CD4^+^ satisfying non-normality or heterogeneity variance, thus Kruskal–Wallis test (including Bonferroni post-hoc correction) was applied

Poorly immunogenic and difficult-to-treat 4T1 tumor was chosen as the object [34]. 4T1 orthotopic tumor model was established to explore the synergistic anti-tumor effect of MnP@Lip and OXA. Figure 5A presented the experimental protocol. The results as shown in Fig. 5B, demonstrated that tumor growth in PBS group was the fastest, and the anti-tumor effect of OXA + MnP@Lip was significantly superior to OXA, MnP@Lip and PBS group. Additionally, results of tumor weight (Fig. 5C) shared a similar trend with Fig. 5B, and this further proved the synergistic anti-tumor effect of MnP@Lip and OXA. As shown in Fig. 5D, the weight change curve of MnP@Lip group almost overlapped with that of PBS group. Moreover, the markers of liver (ALT and AST) and kidney (UREA and BUN) function were within normal ranges and exhibited no significant difference (Fig. 5E). Tumor specimens and H&E staining results of the major organs (liver and kidney) were presented in Additional file 1: Figure S4. These results demonstrated that MnP@Lip possesses good biocompatibility and safety. To clarify the synergistic mechanism of MnP@Lip and OXA, immunohistochemical and immunofluorescence analysis of tumor specimens were performed. Immunohistochemical results of cGAS, pSTING, pIRF3, NF-κB p65, CD206 and iNOS were shown in Additional file 1: Figure S5. The results showed that OXA combined with MnP@Lip nanoparticles could increase the expression of STING pathway related proteins. This suggested that the combination of MnP@Lip nanoparticles and OXA might evoke immune system by STING pathway. The result was shown in Fig. 5F, compared with PBS group, fewer Ki67 expression and more tumor-infiltrating CD8^+^ and CD4^+^ T cells were observed in tumor of OXA + MnP@Lip group. The high expression of Ki67 indicates that tumor cells are highly aggressive and have a high probability of metastasis [35]. The increased CD8^+^ and CD4^+^ T cells proved that MnP@Lip could contribute to recruiting immune cells to tumor microenvironment and boosting adaptive immune response. Overall, the results above led to the conclusion that MnP@Lip could enhance tumor immunogenicity and synergistically boost the anti-tumor efficacy of OXA for 4T1 orthotopic tumor.

Immunogenic MC38 subcutaneous tumor model was also used to explore whether MnP@Lip can synergistically enhance the anti-tumor efficacy of another platinum-based drug, cisplatin (Pt). Experimental protocol was illustrated in Additional file 1: Figure S6A. As shown in Additional file 1: Figure S6B, the tumor growth curve of Pt group almost overlapped with that of PBS group, which indicated that Pt alone has limited anti-tumor efficacy. Delightfully, combination treatment of MnP@Lip and Pt could significantly inhibit tumor growth. Additionally, the results of tumor weight (Additional file 1: Figure S6C) shared a similar trend with Additional file 1: Figure S6B, and this further proved the synergistic anti-tumor effect of MnP@Lip and Pt. Based on above results, MnP@Lip could enhance tumor immunogenicity and synergistically boost the anti-tumor efficacy of cisplatin for MC38 tumor.

**Fig. 6:**
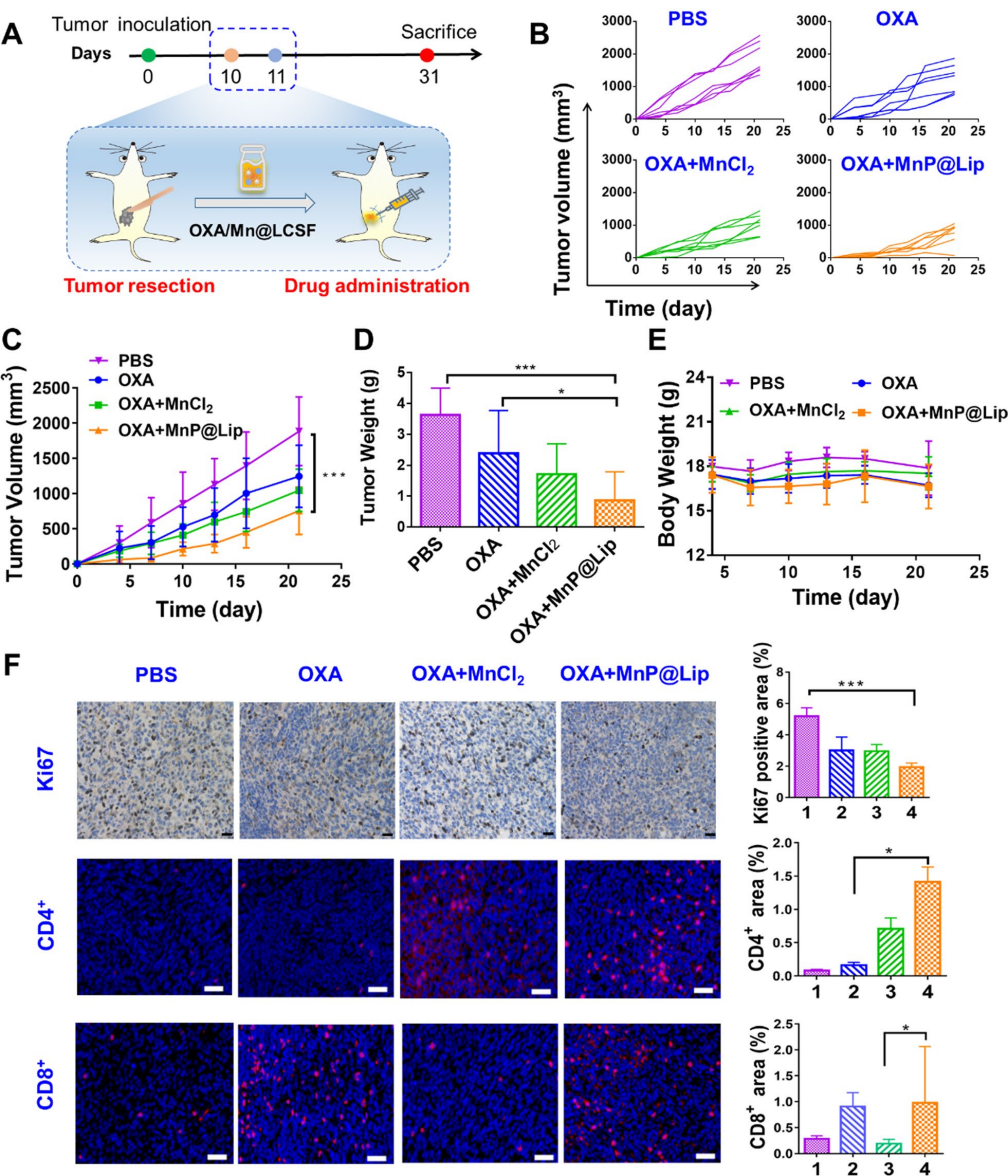
Anti-tumor effect of MnP@Lip combined with OXA in 4T1 incomplete tumor resection model. **A** Treatment protocol of MnP@Lip combined with OXA mediated anti-tumor therapy. **B** and **C** Tumor growth curves of different treatment group (n = 7). **D** Tumor weight and **E** body weight change after receiving treatments. **F** Immunohistochemical and immunofluorescence analysis of Ki67, CD4^+^ and CD8^+^ T cells in PBS, OXA, OXA + MnCl_2_ and OXA + MnP@Lip group, respectively (n = 3). The scale bars are 20 μm, 50 μm and 50 μm, respectively. 1, 2, 3 and 4 represent PBS group, OXA group, OXA + MnCl_2_ group, and OXA + MnP@Lip group, respectively. The data of Figure C and Ki67 satisfying normality, thus One-way ANOVA (including Tukey post-hoc test) was applied. The data of Figure D, CD4^+^ and CD8^+^ satisfying non-normality or heterogeneity variance, thus Kruskal–Wallis test (including Bonferroni post-hoc correction) was applied

The treatment administration of postoperative tumor recurrence is a common but pending clinical problem. Therefore, this study conducted the exploration that whether MnP@Lip was helpful in the treatment of postoperative tumor recurrence. We applied a simple liquid crystal gel formation system composed of soy phosphatidylcholine, dehydrated sorbitan monooleate and tocopheryl acetate for stable drug release, because this system can in situ transform to gel in the site of resected tumor. The result of rheology experiments (Additional file 1: Figure S7) demonstrated that LCFS precursor containing MnP@Lip and OXA could be successfully transformed into gel after injection. Figure 6A presented the experimental protocol. As shown in Fig. 6B and C, tumor growth in PBS group was fastest, and the anti-tumor effect of OXA + MnP@Lip was significantly superior to OXA group and OXA + MnCl_2_ group. Additionally, the tumor weight of OXA + MnP@Lip group was significantly lower than that of the other groups (Fig. 6D). Importantly, one tumor was found disappeared during the autopsy, which further proved the synergistic anti-tumor effect of MnP@Lip and OXA. As shown in Fig. 6E, the comparison results of body weight between OXA + MnP@Lip and OXA group showed that the use of MnP@Lip did not cause additional weight loss, suggesting the good safety of MnP@Lip. Additionally, immunohistochemical and immunofluorescence analysis of tumor specimens were performed to validate the synergistic mechanism of MnP@Lip and OXA. The results were shown in Fig. 6F. Immunohistochemical results of cGAS, pSTING, pIRF3, NF-κB, CD206 and iNOS were shown in Additional file 1: Figure S8. The results showed that OXA combined with MnP@Lip nanoparticles could increase the expression of STING pathway related protein. This suggested that the combination of MnP@Lip nanoparticles and OXA might inhibit tumor recurrence by enhancing STING pathway. Compared with OXA + MnCl_2_ group, the tumor of OXA + MnP@Lip group had fewer Ki67 expression and more tumor-infiltrating immune cells (CD8^+^ and CD4^+^ T cells). This suggested that MnP@Lip with sustained release ability may have a stronger immune activation effect than free Mn^2+^.In addition, the results of the safety analysis indicated that the combination of MnP@Lip nanoparticles and OXA drugs was safe (Additional file 1: Figure S9). Combined use of MnP@Lip and OXA is an effective immunochemotherapy regimen, which may effectively inhibit the postoperative recurrence of 4T1 tumor.

## Conclusion

Overall, this study constructed the simple and cost-effective MnP@Lip nanoparticles to explore the synergistic anti-tumor ability of Mn^2+^ and platinum-based chemotherapeutics, from the aspects of mechanism and phenomenon. Firstly, this study proved that Mn^2+^ can promote the polarization of M0 and M2 macrophages to M1 phenotype, and promote the maturation of BMDC cells. Additionally, upon Mn^2+^ treatment, the STING pathway in other cells was also activated including MLE-12, immune and tumor cells. Finally, given the antitumor results in 4T1 orthotopic tumor model, MC38 subcutaneous tumor model and 4T1 incomplete tumor resection model, the synergistic anti-tumor ability of MnP@Lip and platinum-based chemotherapeutics was verified. This kind of therapeutic strategy combined MnP@Lip nanoparticles with platinum-based chemotherapeutics may provide a novel insight for immuno-chemotherapy.

## Supplementary Information

**Additional file 1.** Additional figures and tables.

## Data Availability

All data generated or analyzed during this study are included in this article.
