# Supplementary material for: A magnetic resonance nanoprobe with STING activation character collaborates with platinum-based drug for enhanced tumor immunochemotherapy
Source: J Nanobiotechnology. 2021 Dec 11;19:415. doi: 10.1186/s12951-021-01158-y (PMC8666035; doi:10.1186/s12951-021-01158-y)
Supplement: Supplementary file 1 — Additional file 1. Additional figures and tables. [file 12951_2021_1158_MOESM1_ESM.docx]

**Supplemental information**

**A magnetic resonance nanoprobe with STING activation character collaborates with platinum-based drug for enhanced tumor immunochemotherapy**

*Jiali Li, ^1^ Shichao Li, ^1^ Yang Li, ^2^ Guanjie Yuan, ^1^ Yaqi Shen, ^1^ Yang Peng, ^1^ Conglian Yang, ^2*^ Zhiping Zhang ^2, 3^ Zhen Li ^1*^*

AUTHOR ADDRESS

^1^Department of Radiology, Tongji Hospital, Tongji Medical College, Huazhong University of Science and Technology, Wuhan 430030, P. R. China.

^2^Tongji School of Pharmacy, Huazhong University of Science and Technology, Wuhan 430030, P. R. China

^3^Hubei Engineering Research Center for Novel Drug Delivery System, Huazhong University of Science and Technology, Wuhan 430030, P. R. China


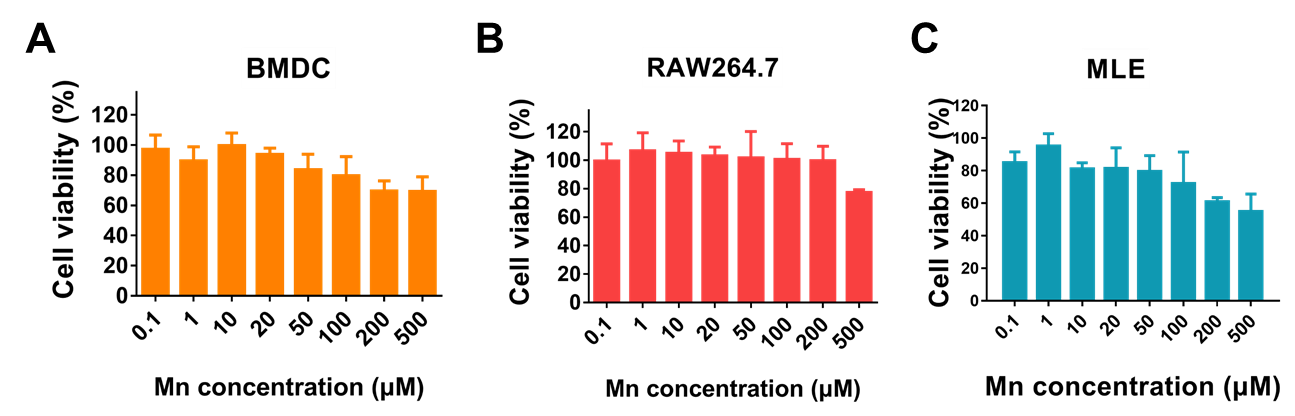


Figure S1. Cell viability of MnCl_2_ on BMDC, RAW264.7 and MLE cells (n=5).


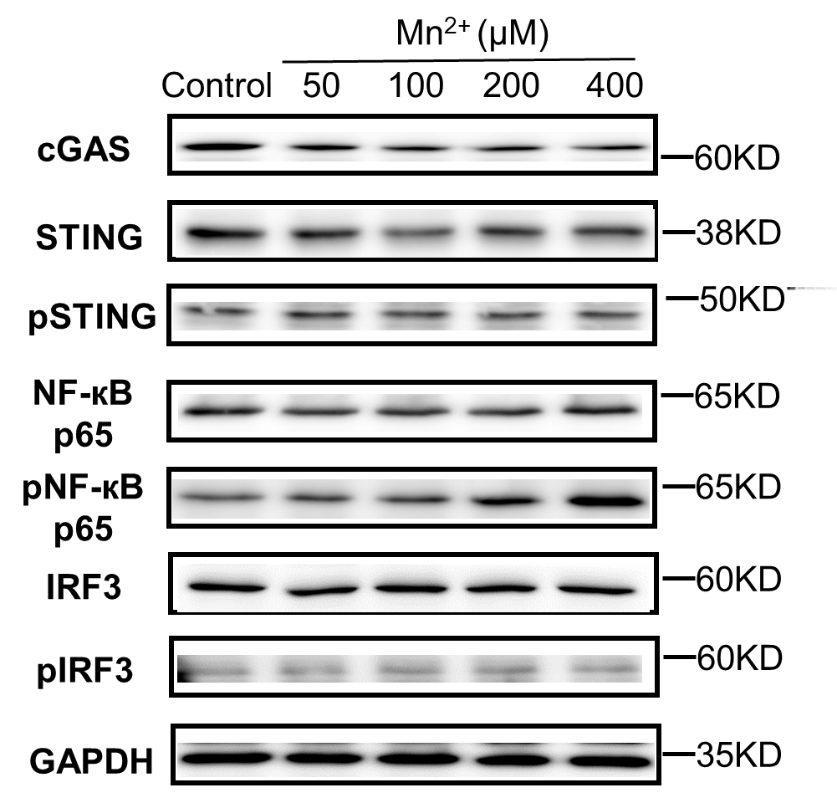


Figure S2. cGAS, pSTING/STING, pNF-кB p65/NF-кB p65 and pIRF3/IRF3 protein expression of BMDC cells that were treated with different concentrations of Mn^2+^.


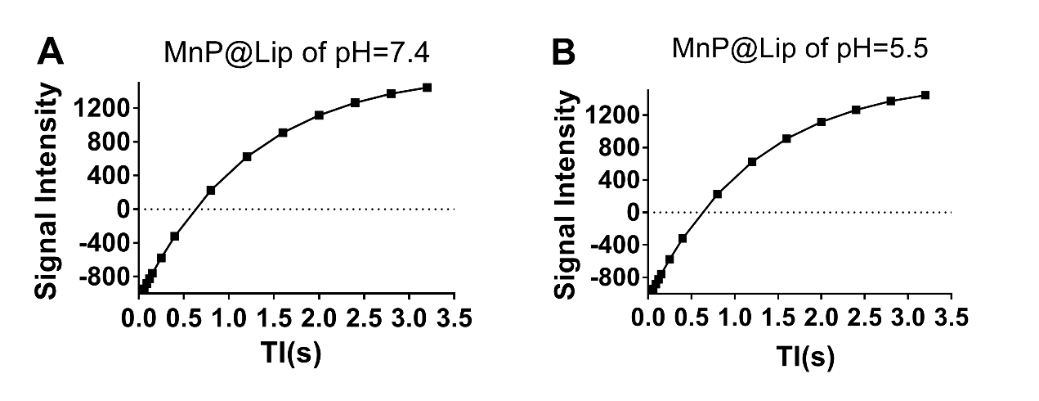


**Figure S3**. Plots of Signal vs inversion times (TI) obtained at 3T MR scanner. (A) and (B) were in pH= 7.4 ABS and 5.5 ABS solution containing Mn concentration of 0.2 mM.


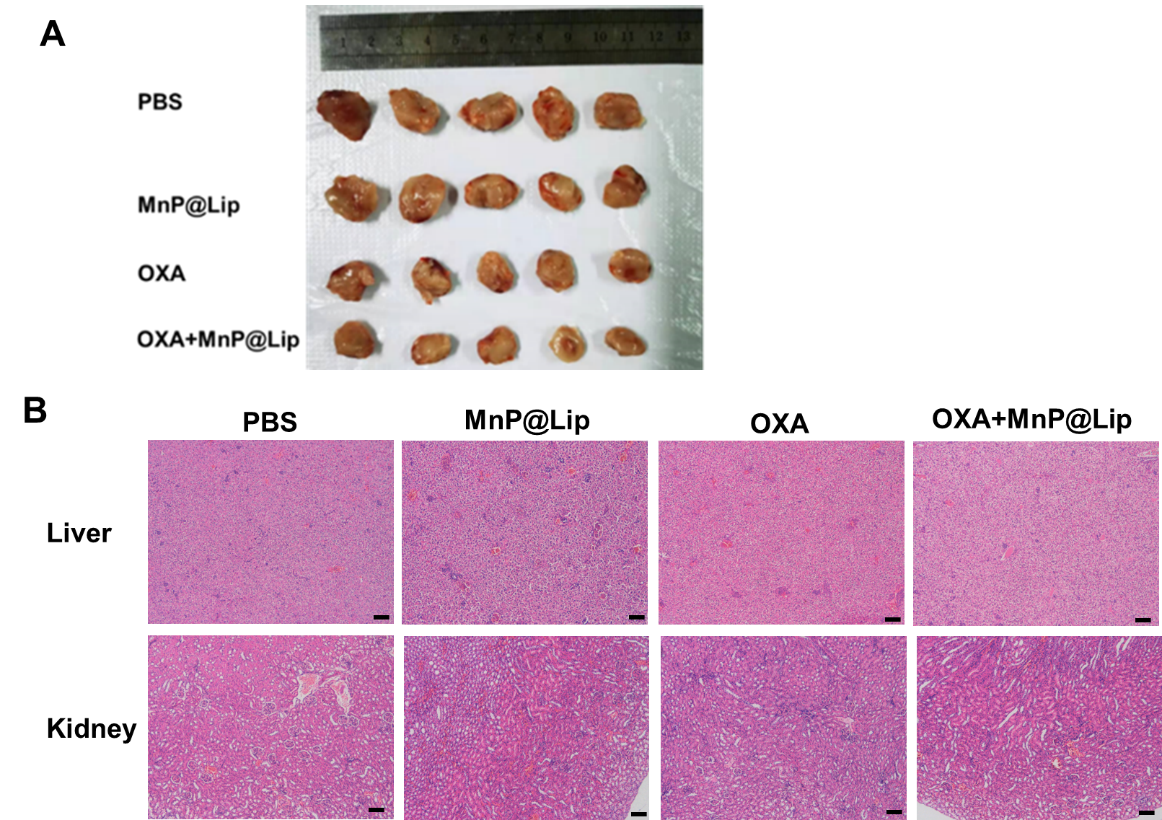


**Figure S4.** Tumor specimens and the H&E staining results of the major organs (liver and kidney) are shown at the end of 4T1 orthotopic tumor treatment. Scale bar: 200 μm.


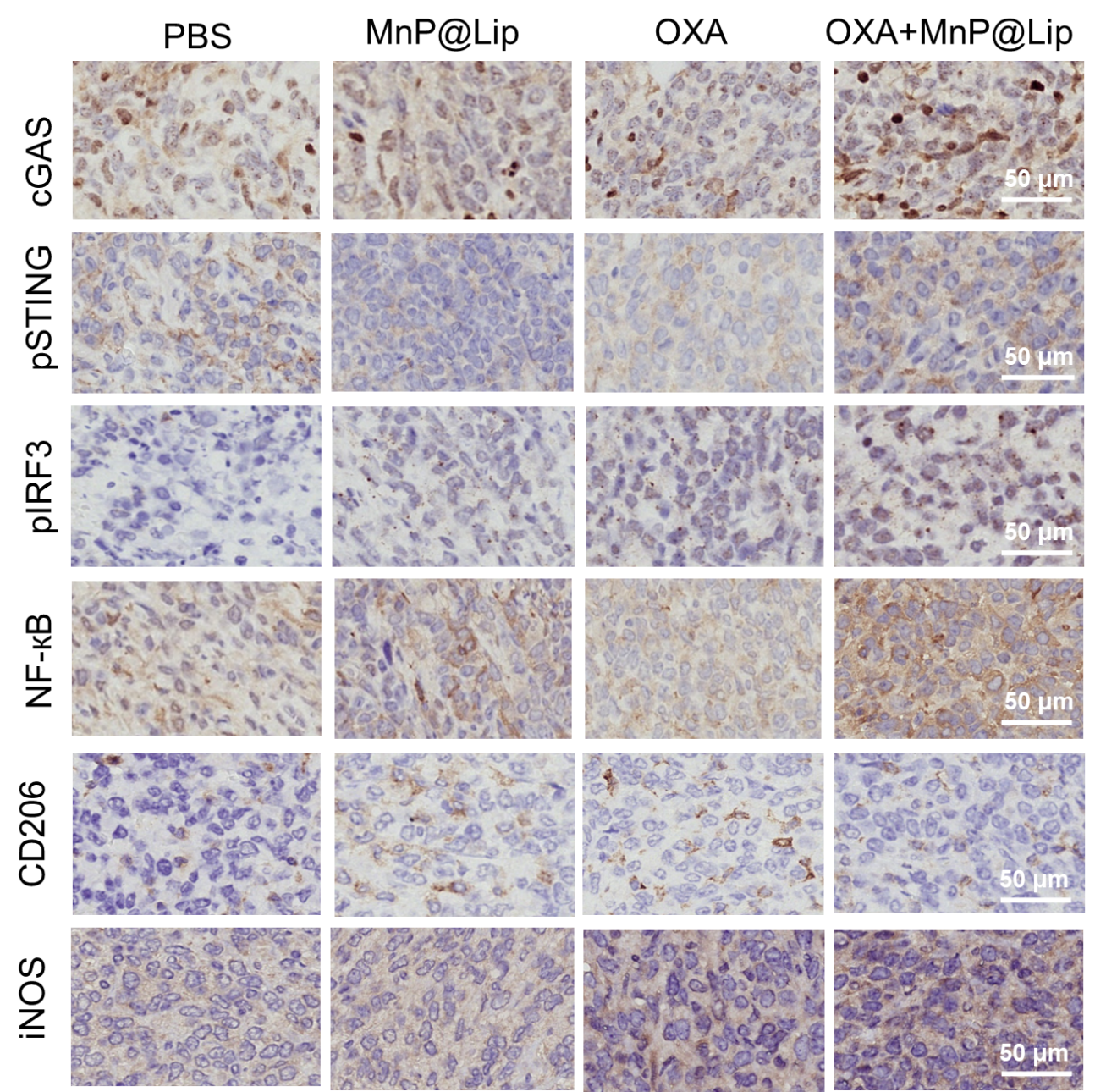


Figure S5. Immunohistochemical of cGAS, pSTING, pIRF3, NF-κB, CD206 and iNOS of tumor tissues in 4T1 orthotopic tumor treatment. Scale bar = 50 μm.


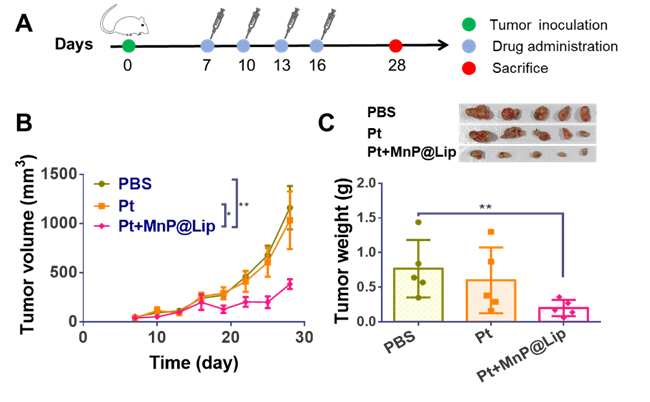


**Figure S6**. Antitumor effect of MnP@Lip combined with Pt in MC38 tumor model. (A) Treatment protocol of MnP@Lip combined with Pt. (B) Tumor growth curves of different treatment group (n=5). (C) Tumor weight after receiving treatments (n=5). One-way ANOVA (including Tukey post-hoc test) was applied to test the data of Figure B and Figure C.


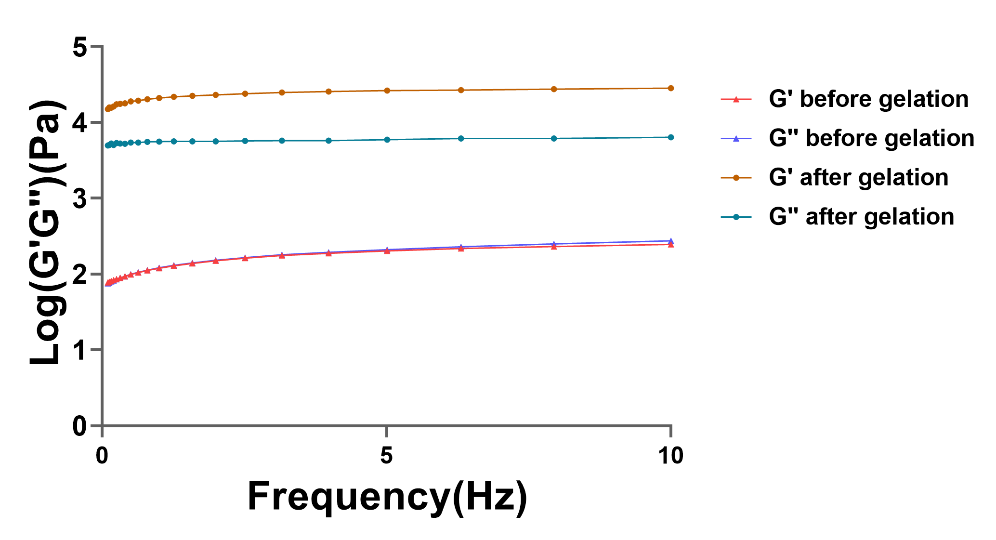


Figure S7. The rheological properties test of liquid crystal gel system (LCFS) containing MnP@Lip and OXA.


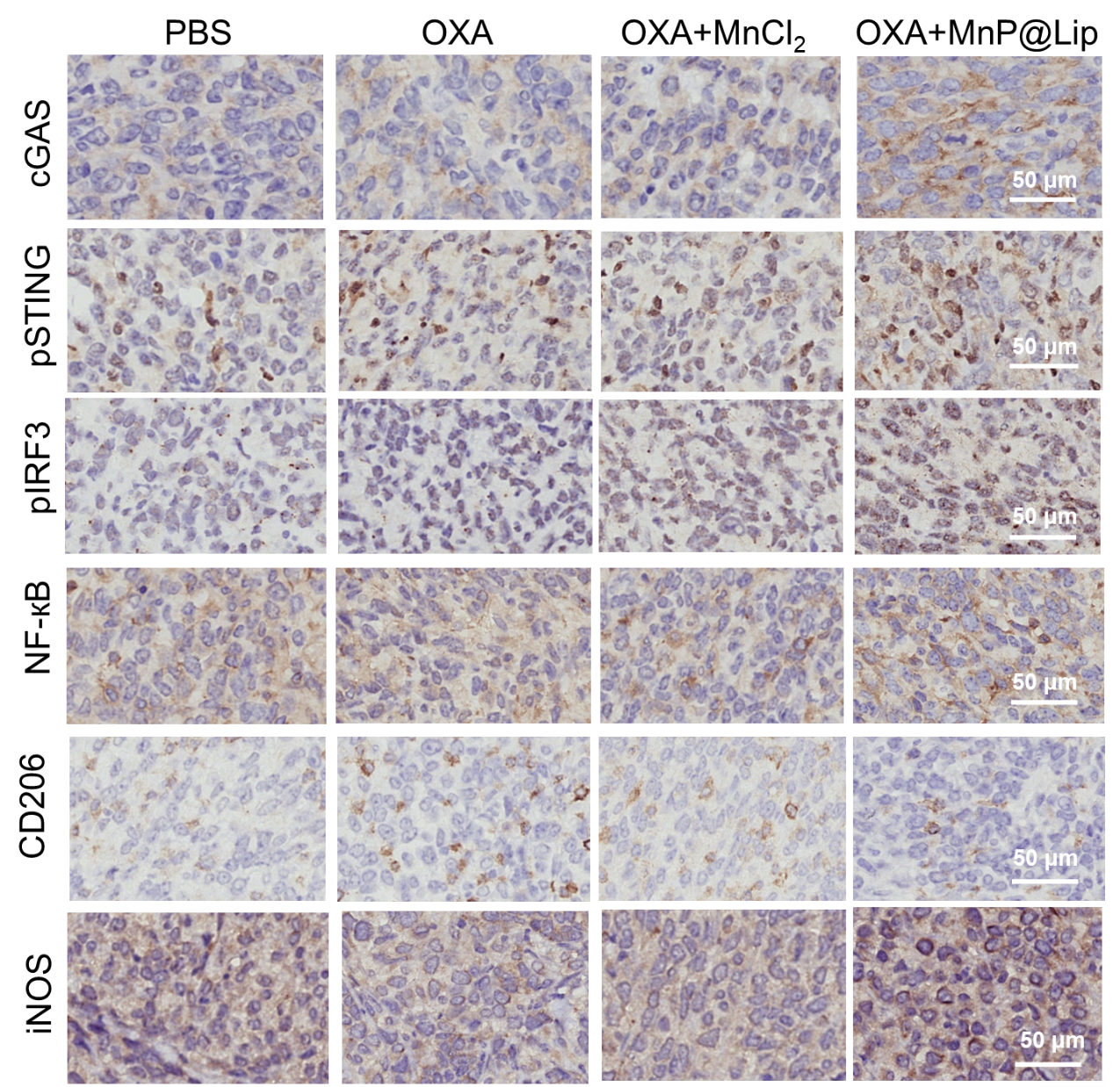


Figure S8. Immunohistochemical of cGAS, pSTING, pIRF3, NF-κB, CD206 and iNOS of tumor tissues in 4T1 postoperative tumor models. Scale bar = 50 μm.


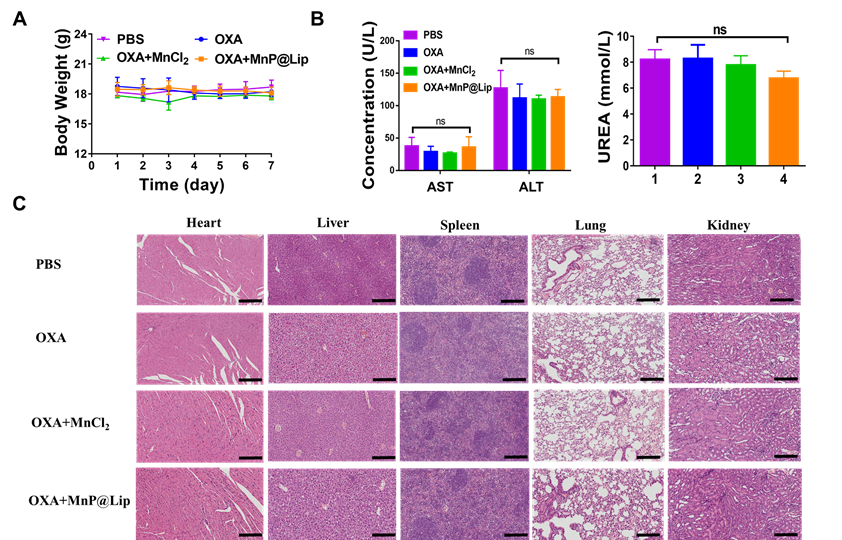


Figure S9. Safety study of combined use of MnP@Lip nanoparticles and OXA. (A) mice weight change during the trial. (B) Effect of drugs on liver and kidney functions. 1, 2, 3 and 4 represent PBS group, OXA group, OXA+MnCl_2_ group and OXA+MnP@Lip group, respectively (n=3). (C) H&E staining results of the major organs at the end of trial. Scale bar: 250 μm.

**Table S1**. Detailed parameters of MRI scanning sequence

|  | TR  ms | TE,  ms | Flip Angle,  Degrees | No.  Averages | FOV  mm^2^ | Matrix | Pixel Size  mm^2^ | Slice Thickness  mm |
| --- | --- | --- | --- | --- | --- | --- | --- | --- |
| T1WI | 398 | 17 | 160 | 8 | 40*40 | 256*205 | 0.156*0.156 | 1 |
